# Anomalous material-dependent transport of focused, laser-driven proton beams

**DOI:** 10.1038/s41598-018-36106-8

**Published:** 2018-12-03

**Authors:** J. Kim, C. McGuffey, D. C. Gautier, A. Link, G. E. Kemp, E. M. Giraldez, M. S. Wei, R. B. Stephens, S. Kerr, P. L. Poole, R. Madden, B. Qiao, M. E. Foord, Y. Ping, H. S. McLean, J. C. Fernández, F. N. Beg

**Affiliations:** 1 Center for Energy Research, University of California, San Diego, La Jolla CA 92093-0417 USA; 2 Los Alamos National Laboratory, Los Alamos, NM 87545 USA; 3 Lawrence Livermore National Laboratory, Livermore, CA 94551 USA; 4 General Atomics, San Diego, CA 92186-5608 USA; 5 University of Alberta, Edmonton, Alberta T6G 2V4 Canada; 6 Physics Department, The Ohio State University, Columbus, OH 43210 USA

## Abstract

Intense lasers can accelerate protons in sufficient numbers and energy that the resulting beam can heat materials to exotic warm (10 *s* of *eV* temperature) states. Here we show with experimental data that a laser-driven proton beam focused onto a target heated it in a localized spot with size strongly dependent upon material and as small as 35 *μm* radius. Simulations indicate that cold stopping power values cannot model the intense proton beam transport in solid targets well enough to match the large differences observed. In the experiment a 74 *J*, 670 *fs* laser drove a focusing proton beam that transported through different thicknesses of solid Mylar, Al, Cu or Au, eventually heating a rear, thin, Au witness layer. The XUV emission seen from the rear of the Au indicated a clear dependence of proton beam transport upon atomic number, Z, of the transport layer: a larger and brighter emission spot was measured after proton transport through the lower Z foils even with equal mass density for supposed equivalent proton stopping range. Beam transport dynamics pertaining to the observed heated spot were investigated numerically with a particle-in-cell (PIC) code. In simulations protons moving through an Al transport layer result in higher Au temperature responsible for higher Au radiant emittance compared to a Cu transport case. The inferred finding that proton stopping varies with temperature in different materials, considerably changing the beam heating profile, can guide applications seeking to controllably heat targets with intense proton beams.

## Introduction

Creation of intense proton beams and their transport are active research topics due to the unique ability of such beams to volumetrically and isochorically heat matter for fundamental studies of non-equilibrium states^1^ as well as materials relevant to conditions in nuclear implosions and the interior of planets^2,3^. Other potential applications of laser-driven beams include generation of neutrons or exotic isotopes^4,5^ and the ion fast ignition inertial fusion concept^6,7^. It has been demonstrated that protons can be accelerated to 10 s of MeV via short-pulse lasers interacting with solid targets. Several different methods have been investigated for ion acceleration including the most studied mechanism target normal sheath acceleration (TNSA)^8,9^, radiation pressure acceleration (RPA)^10,11^, breakout after burner (BOA) ^12,13^ and collisionless shock acceleration^14^. While proton acceleration with various mechanisms has been intensively studied, relatively few works focus on the physics arising during intense proton beam transport in various states of materials.

Early studies on proton beam transport focus on demonstration of isochoric heating of a single solid material^15,16^. For these studies, proton beams were generated through the TNSA mechanism utilizing curved foil targets which provide the beam focusing effect as protons accelerate normal to the target toward the geometric center. When focused, these proton beams became intense enough to heat a matter sample to 10 *s* of *eV*. For instance, (>10^11^) protons generated from a 10 *J* laser were focused to a small spot (<50 *μm*) resulting in isochoric heating of Al foil to over 20 *eV* temperature^15^. Using a higher energy (170 *J*) laser, the brightest emission on Al foil corresponding to temperature of ~80 *eV* was measured^16^.

In proton beam transport, accurate accounting of how protons transfer their energy to matter as they slow down, known as proton stopping power, is one of the requirements to estimate beam projected range and heating profiles in a transport medium. For a non-relativistic proton with initial energy >*MeV* in a metal, the stopping power and total range of the proton are dominated by electronic stopping power caused by frequent inelastic collisions with electrons. For cold materials, probabilistic yet accurate stopping power values exist for charged ions including protons, drawing from broad experimental measurements and theoretical values^17,18^. However, model predictions for ion stopping in more complex states such as warm dense matter (WDM) or dense plasma, being neither solid nor ideal plasma, are uncertain and unverified. Major efforts to describe stopping of charged particles in this regime, a partially ionized medium, have been made via theoretical approaches and numerical calculations. One well-known approach is separately treating bound and free electrons’ contributions to electronic stopping power (B + F model)^19,20^, where fractions of these two populations are determined by plasma state conditions. A more self-consistent standard approach for charged particle stopping, the BPS model^21^, includes perturbative approaches to interactions and dynamic screening instead of simplified methods for the Coulomb energy exchange. With this model, alpha particle stopping range is predicted to be about 20–30% longer compared to most models for deuterium-tritium fusion^22,23^. Applying the local density approximation is another well-known method^24^. Recent calculation (SCAALP)^25^ uses the local-density and average-atom approximations where the inhomogeneous total electron density is taken into account. Simulations using molecular dynamics^26^ have shown that they are reasonably applicable to calculate stopping of ion transport in strongly coupled plasmas. While these approaches can calculate stopping power values, each has limitations and none is valid over a wide range of density and temperature.

Few experiments have attempted to measure proton stopping power in the WDM range because of the paradoxical situation that measurable samples are difficult to create. One measurement of stopping power was made using lower flux protons born from the D, ^3^He reaction to probe a laser-heated sample. The precise energy of these protons allowed for reliable comparison of the stopping calculated by various means but only for a single, high projectile velocity^27^. The experiment was modeled with various models in a recent stopping study and found to be reproduced well using a time-dependent orbital-free density functional theory (TD-OF-DFT) method^28^. A different experimental method was applied to make ion stopping power measurements in laser-heated warm-dense matter, but for a projectile much closer to the expected thermal velocity of the sample, where stopping power is highest and most subject to disagreement between models^29^. The results of that experiment promote advanced stopping models including BPS and T-matrix formulation^21,30^.

Drivers powerful enough to reach WDM conditions may cause the thermodynamic state of the sample to change rapidly, causing difficulties in predicting the behavior of the beam transport. In this case, responses of the matter and the beam to one another need to be taken into account simultaneously. Self-consistent description of intense proton beam dynamics with collective effects has been studied using PIC simulations with a B + F stopping model by this group^31,32^ and PIC with Monte Carlo stopping^33^. However, these transport studies with simple stopping models are limited to a few materials and would benefit from experimental findings in relevant conditions for various materials.

In this paper, we present experimental and simulation results of proton beam transport in different solid materials with a proton beam with a fixed source. Presented first are measurements of thermal emissions due to focused beam heating. To address the experimental questions such as the cause of dependence of the emission characteristics upon transport materials and heated temperature at the rear of the target, simulations using a particle-in-cell code were carried out. Simulations of the time-dependent source are described in the second Results subsection, followed by transport simulations using the same code. Details of the experimental and computational setups are described in Methods.

## Results

### Experimental measurements of 68 eV emission from the target rear

**Figure 1:**
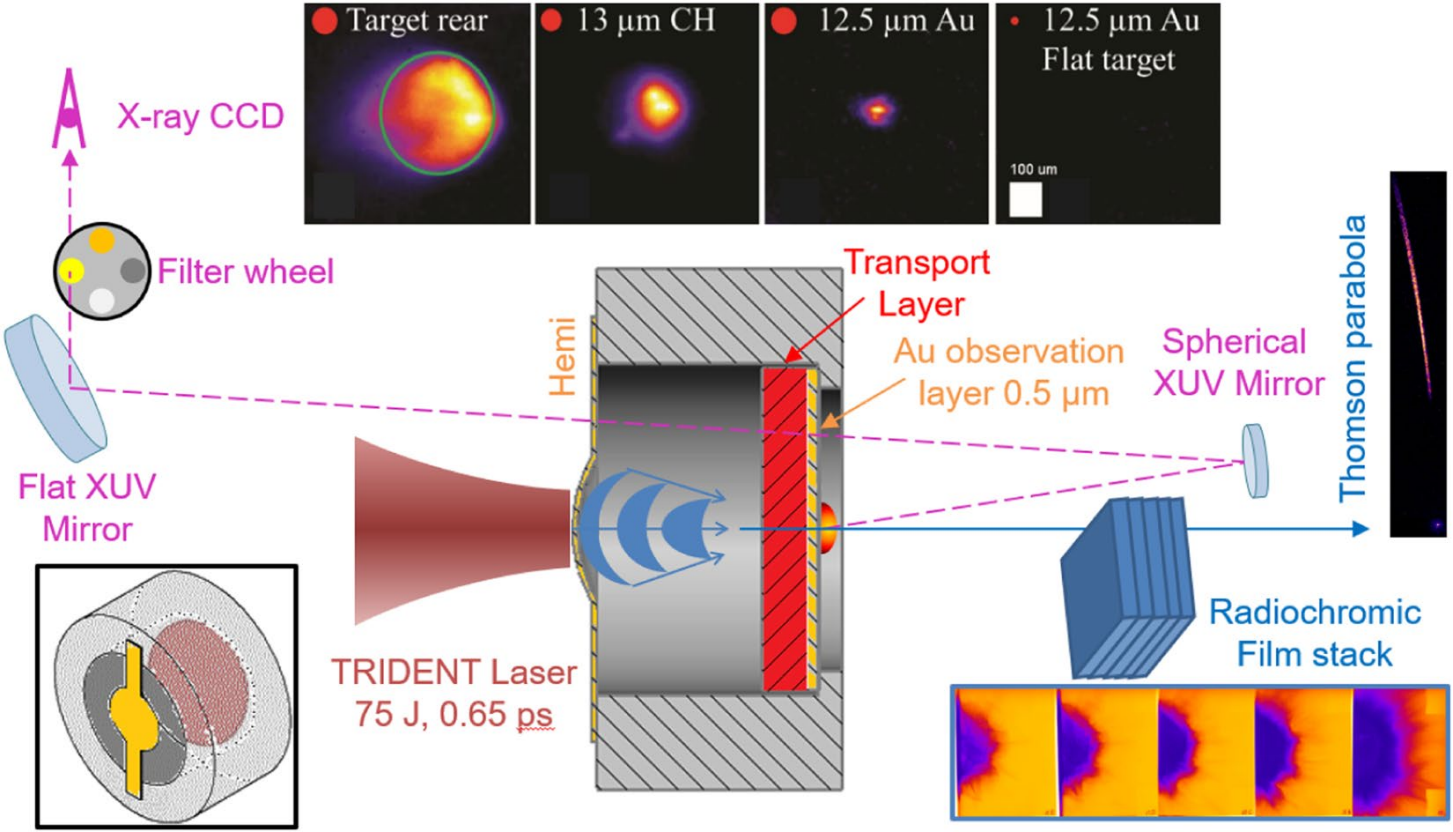
Diagram of experimental setup; the overall target is shown in lower left inset. The Trident short pulse laser is incident on a flat or a curved (partial hemisphere) front foil to generate the proton beam. The XUV system images thermal emission on the Au layer behind the transport foil; several examples are shown (top sequence). The red circle indicates the laser spot size on target, while the green circle is the Au hemi size. Particles are recorded nearly along the target rear normal with film (bottom series) and Thomson parabola spectrometer (right inset).

A schematic diagram of the experiment and example data types are shown in Fig. 1. The TNSA proton beam generated by irradiation of the 100 TW-class Trident laser onto a Au partial-hemispherical target (“Au hemi”) with radius of curvature *r*_*c*_ = 300 *μm* was focused as discussed below. At a distance ~500 *μm*, ~1.7 × *r*_*c*_, the focused proton beams entered then transported through solid foils having a broad range of atomic number, Z, including Mylar, Al, Cu or Au with different thicknesses and a thin Au witness layer.

The transport foil thickness for the different materials was chosen to have equivalent stopping range in cold matter using the NIST data of proton stopping^18^. Two series of foils were chosen. First, Mylar (CH), Al, Cu and Au had a thickness of 50 *μm*, 30 *μm*, 15 *μm* and 12.5 *μm*, respectively, all chosen to range out 1.7 ± 0.1 *MeV* protons. Second 13 *μm* Mylar and 12.5 *μm* Al were chosen to range out 0.9 ± 0.1 *MeV* protons. For all materials, 0.5 *μm* Au layer was deposited on the rear of the transport foil to be a consistent surface for thermal emission measurements. An aluminum cylinder was fabricated to fix the distance between the Au hemi and target rear for all target types. Hot electrons generated by the laser interacting with the Au hemi can spread through the structure and reach the rear, potentially affecting the measurements. To reduce this effect, the structure was made wide (R = 550 *μm*) and the Au hemi was connected to it with thin support strips.

As a main measurement of beam transport, an extreme ultraviolet (XUV) imaging system imaged thermal emission from the back side of the Au layer in two dimensions. Since Planckian radiation intensity sensitively varies depending on the temperature of a material, the time integrated brightness of radiation is an indicator of the surface temperature of a target with caveats discussed later.

There are two important reference cases in the XUV dataset to consider. First, targets with no foils behind the Au hemi were shot, and bright emission was observed spread over the 300 *μm* Au hemi rear. The proton source was also measured in these shots. Second, a target consisting of a flat 10 *μm* Au target foil and Au transport foil was irradiated with the laser fully focused on the front foil to achieve highest possible intensity. The XUV emission from the rear Au foil in this case was undetectable. This indicates that the heating from an unfocused proton beam and from any electrons that may have transited the Al structure were insufficient to heat the rear Au surface to the point of considerable 68 eV emission. Note that a typical unfocused TNSA proton beam with 50 *μm* source size and expansion cone angle of 20 degrees would overfill the 500 *μm* diameter transport foil.

For all other shots the laser irradiated target was a Au hemi. Contrary to the flat target case the XUV emitted from the surface behind the transport foil was confined locally, even smaller than the laser focus in some cases, and filters were necessary as the emission brightness was at least 3 orders of magnitude greater. This is due to the focused proton heating. Collected XUV images from different targets are shown in Fig. 1 (top series). Most interestingly, for different transport layers, the emission characteristics changed including emission size and brightness. For instance, emissions from a target with low Z material such as CH and Al were consistently larger (>factor of 2) than ones with Au and Cu.

**Figure 2:**
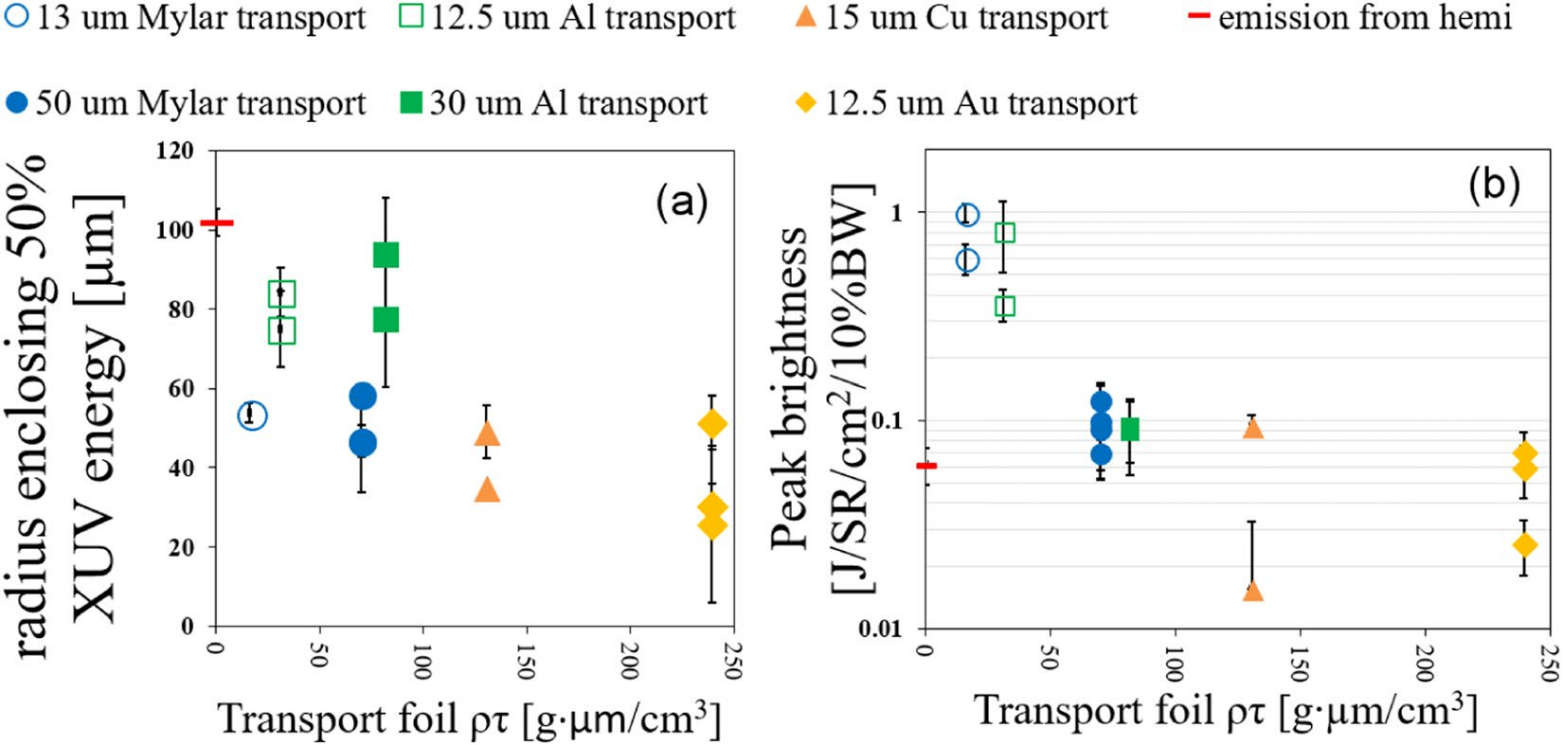
XUV emissions observed from the Au layer behind a transport foil of different materials and thicknesses. Each data point represents a single shot. Areal density of target versus XUV emission size (**a**) and XUV peak brightness (**b**). The red dash point plots the case of a target with neither Au nor transport foil so that the rear of the Au hemi was visible. The error bars in (**a**) are the difference between the vertical and horizontal FWHM. The error bars in (**b**) include the laser energy variation and the range of two different peak analysis approaches.

Details of emission results for all transport foils are shown in Fig. 2. Two plots present the emission size (a) and peak brightness (b) as a function of areal density, *ρτ*. Interestingly, all the targets with Al had a much wider emission size than targets with any other transport layer material or thickness, including those with higher atomic number and higher areal density. The largest emission size (~85 *μm* radius enclosing 50% XUV energy) from a target with Al is more than twice the size (~35 *μm*) of the Au foil cases. In contrast to this strong material dependence, emission size is seen to be weakly sensitive to transport layer thickness comparing data points with the same material, but different thicknesses such as Al (open vs. closed green squares) and CH (open vs. closed blue circles). XUV peak brightness versus target *ρτ* is plotted in Fig. 2(b) and is seen to have even stronger dependence than emission size. In this comparison, the dependence of emission brightness on target material (*Z* or *ρτ*) is not straightforward, but low Z materials (CH and Al) tend to show brighter signal than Cu and Au. A clear trend is that targets with the thin layer have much brighter emission (~4×) than their thicker counterparts having the same material.

Discrepancies exist between these experimental results and predictions of simple existing models. For example, Monte Carlo calculations^34^ predict lateral straggling that increases with thickness but has extent of only several *μm* for the thicknesses investigated with thick Au being the most extreme at ~12 *μm*. As seen in the data above, the heated sizes vary by a much wider range, from 30 *μm* to 80 *μm*. Also, wider emission size for low *ρτ* material is contradictory to Moliere nuclear scattering formulas^35^ where protons traveling in high *ρτ* material have wider scattering angle. Further, one might speculate that Cu and Au, having higher room temperature thermal conductivity and more free electrons than Al and CH, would experience greater lateral spreading of the heat deposited via proton stopping, heating a wider portion of the Au emission surface. However, the data oppose all these trends with Cu and Au cases having the smallest emission regions. For these results, it can be concluded that transport for dense proton beams cannot be explained by only cold stopping and scattering models. Particle-in-cell (PIC) computational modeling of the proton beam transport with fields and an advanced model for proton electronic stopping power^31,32^ was conducted to aid in the interpretation of experimental observation as described in the following section. It will be shown that the nonlinear dependence of emissivity versus temperature must also be taken into account in understanding these experimental results.

### Simulating a parameterized proton beam source using particle-in-cell LSP

Simulation of the experiment was conducted using particle-in-cell code LSP^36^. Simulating the configuration at full scale would require ~40 ps simulation run time at great computational expense so that the entire beam including low energy ~1 *MeV* protons can traverse the transport and witness foils positioned 450 *μm* behind the hemi. Moreover, this simulation would need to be repeated for different transport materials. Thus, to efficiently use computational resources, the problem was divided into two steps. First, proton beam generation and drift was modeled and compared to the measured spectrum. The resulting proton beam source was then used in a second series of transport simulations. Details of the simulations are given in the Methods section.

**Figure 3:**
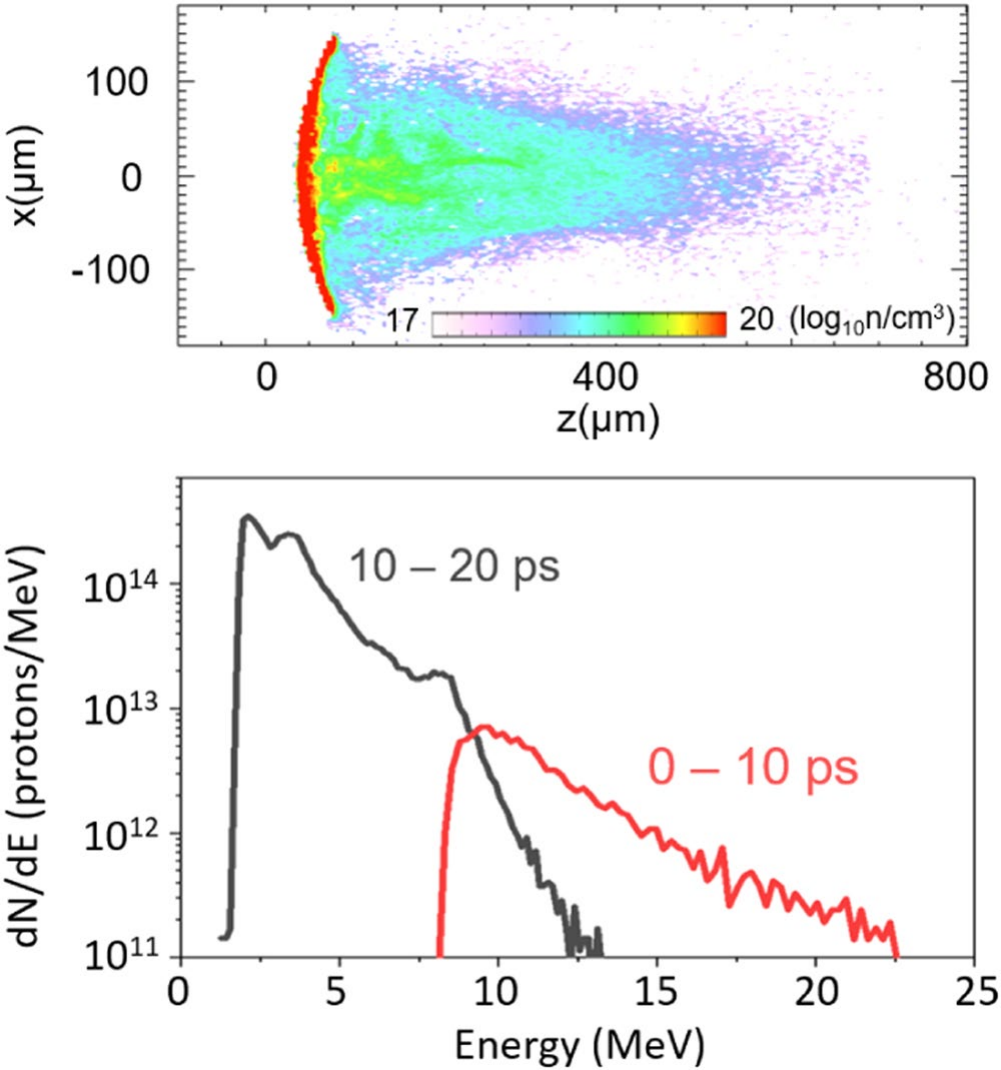
(**a**) Snapshot of protons generated from a partial-hemi target in the PIC source simulation. The energy spectrum of protons collected at *z* = 400 *μm* in the first or second 10 *ps* period (**b**).

The source simulation was performed by injecting a population of electrons with parameters based on the measured laser qualities at the laser interaction location on the hemi. The target geometry was reconstructed in 2D Cartesian coordinates and a kinetic hydrogen species was initialized on the hemi rear. Electrons quickly spread out from the front side of the hemi target and expanded into the vacuum away from surfaces, driving a typical TNSA sheath field. At the rear side of the hemi, this field was strong enough (>1 *TV*/*m*) to accelerate protons to *MeV* energy. Those protons initially accelerated normal to the target (moving toward the target geometric center) then expanded transversely by radial fields generated in the beam. Figure 3(a) presents a snapshot of proton density at 20 ps.

**Figure 4:**
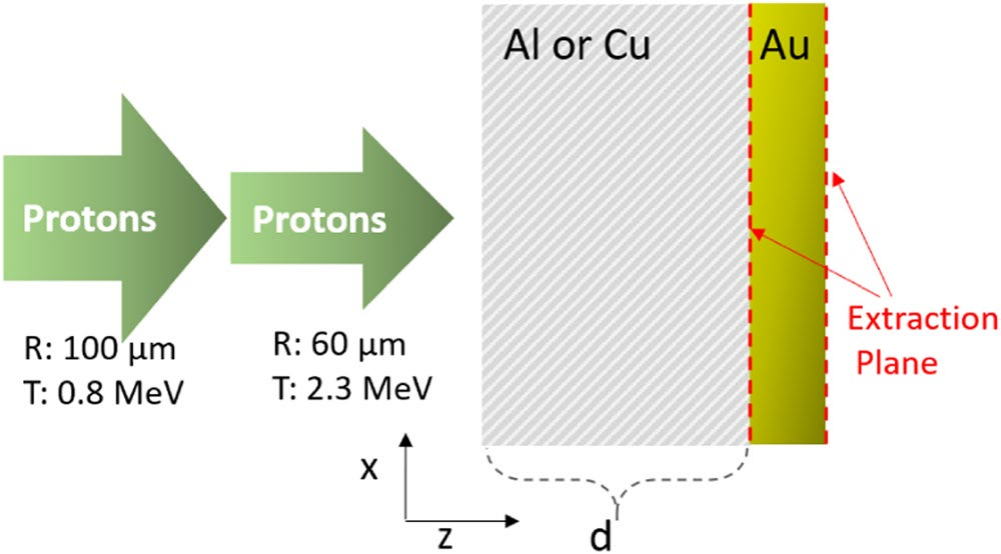
Domain schematic of the transport simulations. Two proton beams with different parameters are sequentially injected inside a transport layer whereby standoff distance from the Au layer, *d*, varies with material; Al case: 30 *μm* and Cu case: 15 *μm*. Proton beams injected earlier and (later) are characterized with slope temperature of 2.3 *MeV* (0.8 *MeV*), pulse energy of 0.45 *J* (0.75 *J*), pulse duration of 7 *ps* (13 *ps*) and gaussian spatial profile with 60 *μm* radius (flat profile with 100 *μm* radius).

Critically, it was observed from the proton generation simulation that the proton source could not be well parameterized with single fixed values. Two main characteristics of the proton beam are (i) proton energy distribution varied with time and (ii) the beam spatial profile at the extraction plane was different for differing proton energies. Figure 3(b) shows the time integrated proton energy spectrum for two time intervals (time integrated up to 10 ps and from 10 to 20 ps) measured at the extraction plane (*z* = 400 *μm* where *z* is the longitudinal axis). Fast moving protons (the early time group, arriving during the 0 to 10 *ps* interval) present a higher slope temperature, while those arriving later (10−20 *ps*) had a lower slope temperature. The second characteristic of the proton beam is that higher energy protons are focused to a small waist when they reach the extraction plane, whereas lower energy protons are uniformly spread over a larger radial area. For these reasons, it was decided that the beam entering the transport layers could not be represented by a beam with a steady-state, single characteristic spectrum, nor single size. Instead, the early and late protons were fit to two beams with duration, total energy, size and characteristic slope temperature given in Fig. 4 and its caption.

### Hybrid PIC simulations of particle transport through self-consistently heated transport layers

To study transport in the foil layers, one key factor is particle stopping power, which determines how much energy particles lose and ultimately where they stop in the medium. The same PIC code was used in a different mode to study transport in the secondary target. While the PIC algorithm precludes explicit binary collisions, quantum and atomic-scale effects cannot directly be included. However, this code can allow new understandings into intense beam transport due to an included dynamic proton stopping power module^31^ that updates according to the thermodynamic state of the material locally. Cases were also run with cold stopping^18^ for comparison. Two materials were chosen, Al and Cu, since in the experiment these transport materials exhibited dramatically different features of XUV emissions. Two proton beams were injected sequentially into the transport material with beam input parameters according to the representative fits discussed in the previous section. The total energy in both beams is 1.2 *J*, which is about 1.5% of the laser energy used in the experiment. Details can again be found in Methods.

**Figure 5:**
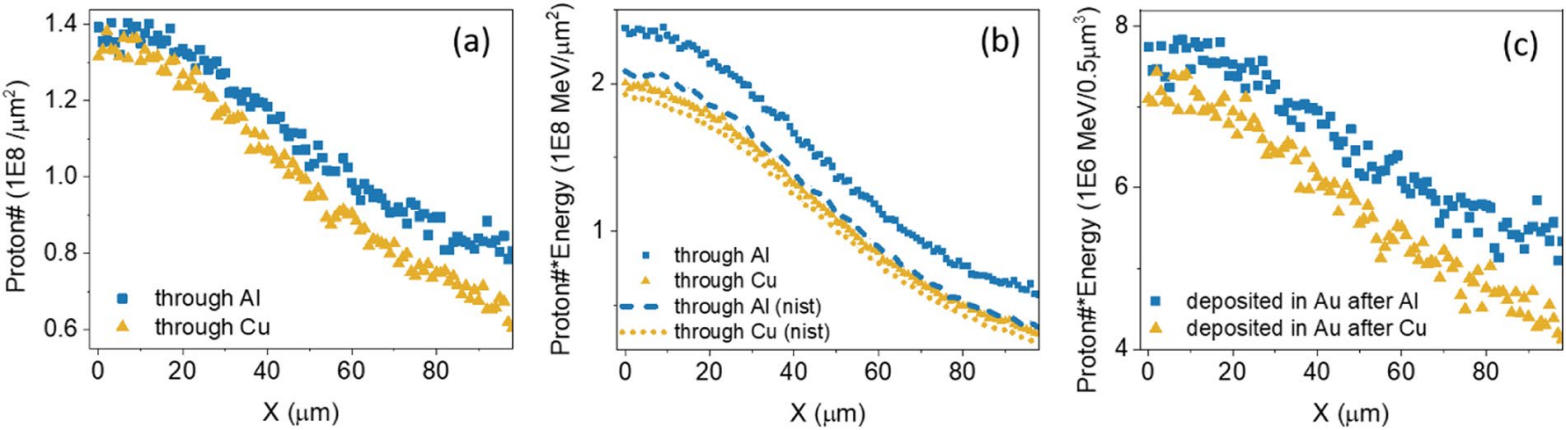
Time-integrated proton number (**a**) and proton energy (**b**) fluence profiles in the x-axis measured at the exit of the transport layer (30 *μm* Al or 15 *μm* Cu). The bin width for both dimensions is 1 *μm*. (**b**) Includes proton energy fluence results obtained using the dynamic stopping power and cold stopping power (NIST) for comparison. (**c**) Shows the time integrated proton energy fluence that is deposited in the Au layer (up to 0.5 *μm*).

Figure 5 shows results of the transport simulations. Proton numbers and proton energy fluence counted at the first extraction planes, time-integrated until all protons leave the transport layer (Al or Cu) are shown in Fig. 5(a,b). From Fig. 5(a), it can be seen that more protons pass through the Al than the Cu layer. Interestingly, the difference is greater far from the central beam axis, in the lateral wings where mostly lower energy protons were injected. Similarly, a higher proton energy fluence is shown in the case of the Al transport layer. In contrast, when the fixed stopping power (cold stopping from NIST) is applied for both Al and Cu layers in simulations, energy fluences for both cases are nearly overlapped (dashed lines). Because the thicknesses (30 *μm* and 15 *μm*) of the two layers were determined by cold stopping range (for a proton of 1.7 MeV), similar energy fluences are expected. Varied energy fluence in the dynamic stopping simulation indicates that protons traveling in Al lose proportionally less energy than the Cu case as proton stopping power changes with the thermodynamic states of the transport layer. This result informs us that choice of a dynamic, or at least non-room-temperature stopping power model is necessary to reproduce the differences in proton transport observed in the experiment.

Total proton energy fluences deposited in the Au layer are found by the energy lost by particles between the Au entrance and exit extraction planes. Particles that were counted in the entrance extraction plane but not the exit plane were assumed to deposit all their energy in the entrance bin. The deposited fluence curves are shown in Fig. 5.

**Figure 6:**
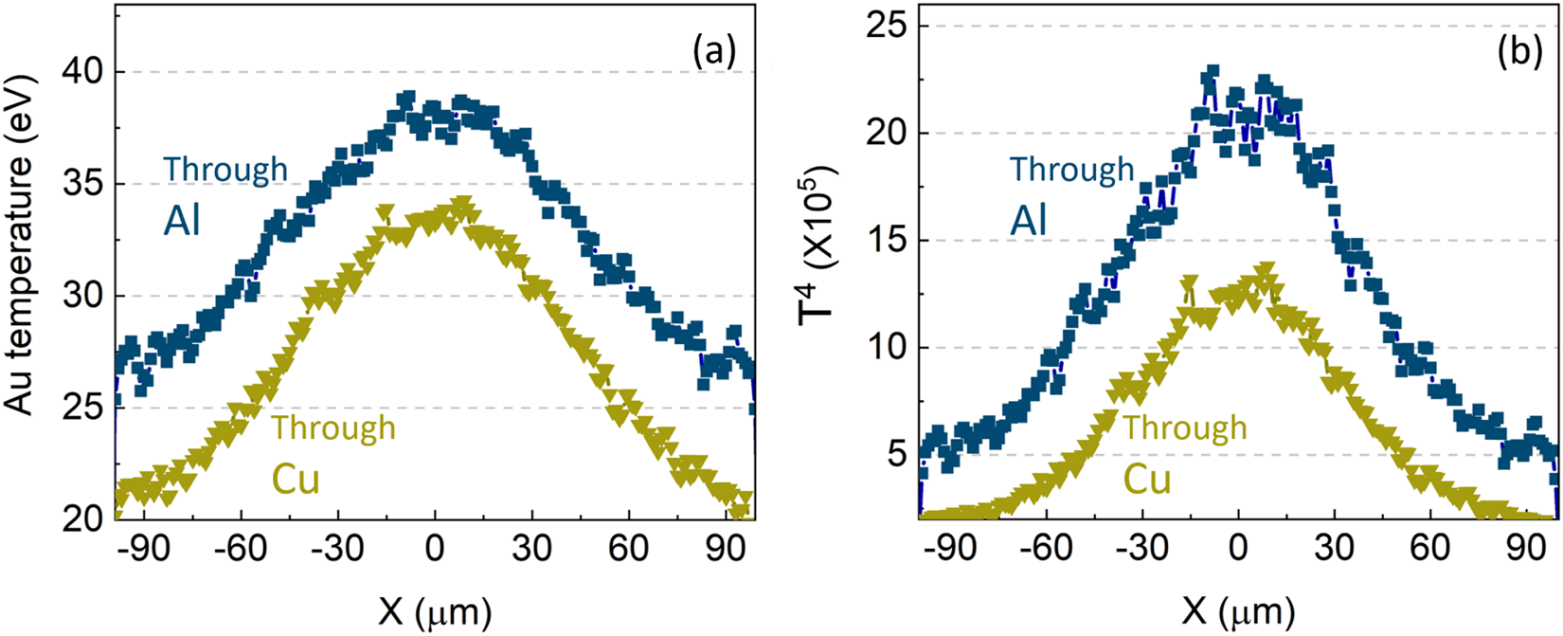
Spatial temperature profile (**a**) and the fourth power of temperature (**b**) of Au target at 0.5 *μm* depth for both Al and Cu cases.

Here, the heated temperature of Au can be estimated using the deposited energy fluence. For instance, energy fluence through the Al layer is ~7.5 × 10^6^ *MeV*/*μm*^2^ at the first bin (0–1 *μm*) in x-axis corresponding to a volume of 0.5 × 1 × 1 *μm*^3^. Using the volumetric heat capacity of Au, 2.43 × 10^−12^ *J*/*Kμm*^3^, the calculated Au temperature is 40 eV. This estimation is similar to the simulation result as shown in Fig. 6(a) where peak temperatures at a central region for both Al and Cu cases are ~38 eV and 33 eV respectively.

Using XUV emission brightness as a temperature diagnostic has caveats. A radiating blackbody with temperature 14 *eV* has a peak in spectral radiance at 68 *eV*. This means that the response of the imaging system is not linear and can exaggerate the cooler regions further out from the spot. Additionally, emittance is strongly dependent on temperature. The Stefan-Boltzmann law describes the total power of black-body radiation depending on the object’s temperature with the relation *j* = *σT*^4^, where *j*, *σ* and *T* are respectively the radiated emittance, Stefan-Boltzmann constant and temperature. While this does not hold true for a single photon band, its scaling shows how a temperature difference of just 5 *eV* can lead to a large difference of radiated emittance. As shown in Fig. 6(b), the fourth power of each temperature of 38 and 33 becomes ~2.1e6 and ~1.2e6 respectively. The ratio of these values is lower than experimental measurement where >2× higher XUV brightness was measured from the Al case compared to the Cu case. However, this modeling treatment reproduces the direction of the trend of XUV brightness from the two different materials.

An important feature is the relatively gradual lateral temperature gradient in the Al case compared to the Cu case. This trend is clearly seen particularly for the outer part in the transverse direction (x-axis), leading to >2× higher emission brightness for the Al case. The XUV imaging has a limitation for measuring emission brightness because the target emitting XUV expands rapidly, resulting in the reduction of absolute brightness. For example, previous work using LASNEX (radiative-hydrodynamics code) simulation^37^ predicted that the XUV brightness corresponding to a Cu target temperature below ~20 *eV* is hardly detectable. Considering this limit, it can be inferred that the Al case has a broader area over which XUV emission can be detected. For Al and Cu layers, ranges (X-axis) for temperature above 30 eV are 130 *μm* and 70 *μm*, respectively; for temperature above 25 eV they are 200 *μm* and 120 *μm* respectively. This difference in size of the signal in simulations has the same trend observed in the experiment where emission size (*R* ~ 80 *μm*) for Al target is about twice that from a Cu target. Note that the beam central area is heated by two characterized beams, but only the second beam (low energy protons) is involved in heating of the wings. Thus, an inference can be drawn that low energy protons are responsible for the clear distinction in heating profile at the wings for the two different material cases.

Additionally, the induced magnetic field during proton beam transport was measured in the simulation to see if the magnetic field had any influence on beam profiles in different materials. However, the proton beam was not intense enough to produce high magnetic fields, (the maximum magnetic field is below 10 Tesla for a given proton beam) and it cannot affect the beam transport in the low thicknesses used.

## Discussion

In this work, the experiments showed that the size and brightness of XUV emissions caused by proton beams vary by a factor of >2 with the target material, demonstrating an unexpectedly strong dependence of proton beam transport on materials.

Simulation modeling of the experiments showed that protons moving through an Al transport layer heat the rear of Au to higher temperature resulting in higher radiated emittance compared to a Cu transport case. This result is consistent with the experimental measurement showing the different brightness of XUV between two material cases. Higher temperature over an entire area of Au for the Al case helps explain the brighter and wider XUV emission size seen from the experiment. These results provide an understanding that the variation of stopping power with temperature in different materials can considerably change the beam heating profile in targets. This effect turned out to be dominant rather than other effects such as beam scattering and induced weak magnetic field for the pertinent proton beam density.

In the range of target temperature (<40 eV) encountered in this work, change of stopping power is relatively considerable only for protons having energies below 1 MeV. Thus it can be inferred that low energy protons played an important role in varying heating profiles in different materials with given thicknesses. Currently, stopping power models for low energy protons in CH or Au materials are limited and unvalidated, inhibiting the range of our investigation. More accurate stopping models in this regime would help us carry out further investigations of the physics of intense beam transport in solids.

## Methods

### Laser, targets, and diagnostics

The experiments were conducted using the Trident laser facility at Los Alamos National Laboratory. Chirped pulse amplified laser pulses delivered an energy of 74 ± 7 *J* on a target in a duration of 670 ± 50 *fs* with an incidence angle of 22.5°. The spectral phase and pulse shape of the laser have been carefully characterized by the facility^38^ through frequency resolved optical gating (FROG). Measurements were taken with a spectrometer and a FROG on most shots in this experiment of the pulse leaving the compressor as well as the pulse reflected from the target. The laser spot was characterized by relaying an image onto a CCD at low laser power. The spot at the target apex plane contained 50% of the laser energy within 65 *μm*, achieved by deliberately defocusing to uniformly cover a large area of a target with the desired outcome that doing so can aid beam focusing by the TNSA mechanism with a curved target^39,40^. The intensity was 3 ± 0.5 × 10^18^ *W*/*cm*^2^. No attempt was made to measure or mitigate spatio-temporal coupling effects^41^ which, if present, would equally affect all shots. Similarly, non-uniformities in the defocused laser spot, which are known to manifest in the proton beam^42^, are presumed to affect the proton source equally for all shots. The 10 *μm* Au hemi was chosen as the proton source target because experience has shown it to be reliable and relatively free from instability-induced structures in the proton beam profile.

To measure the energy spectrum of generated protons, a stack of radiochromic films (RCF) and Thomson Parabola (TP) were used on each shot; for the electron spectrum, a magnetic spectrometer was fielded just below the TP line of sight. In the XUV imaging system^37^, the first optic was a spherical mirror with 50 *cm* radius of curvature, *r*_*curv*_, consisting of 21 pairs of Mo_2_C and Si layers. It was placed 20 degrees off the target rear normal at a distance of 27.6 *cm*, where it reflected 68 *eV* light to a second, flat mirror in a turning chamber and imaged it onto a CCD filtered with Al.

### Simulations

Modeling was conducted using the implicit, hybrid, particle-in-cell code LSP^36^. The simulation method used here to model the TNSA mechanism is to inject relativistic electrons that are representing those accelerated by laser interaction with a target^40,43^. For this injection, electrons of *T*_*e*_ = 0.5 *MeV* distribution with 650 fs duration were chosen, based on the fast electron spectrum measured with an electron spectrometer during the experiment as well as a reasonable agreement to the Beg and Ponderomotive scalings^44,45^ for the Trident laser parameters. Energy coupling from laser to electron was assumed to be 20%^46^. In a 2D cartesian simulation, both Au ions and electrons in a partial hemi with the same size as the real Au target are treated as a fluid, and a kinetic hydrogen layer is positioned at the rear of the hemi target.

In the proton transport simulations, both ions and electrons for materials are set up as fluid where their ionization and heating capacity are updated from the equation of state tables produced by PROPACEOS^47^. Although PIC simulations have limits to provide accurate interpretation of quantum phenomenon for warm dense matter, many effects for proton beam transport are self-consistently modeled with dynamic stopping calculation which is based on matter’s local thermodynamic states (density, temperature, and charge distributions.) Also fields evolution is determined by both beam current and matter’s local resistivity change. Electrons co-moving with protons in the TNSA beam have an energy of only several keV, stopping at the target front surface. As their impact on the transport is negligible, only protons were sent to the target in these simulations. Once protons travel through a transport layer (Al or Cu) up to distance *d* = 30 *μm* and 15 *μm* respectively, the proton data including particle energy (3 dimensions in velocity space) and positions in 2D are recorded in the extraction plane and re-injected from that plane into an Au target. The last extraction plane is positioned at 0.5 *μm* inside the Au as following the experiment where 0.5 *μm* Au layer was coated at back surface of transport foil. In these simulations, dynamic proton stopping power^31^ that is updated based on its thermodynamic state at each time step was applied for Al and Cu. For Au medium, only cold stopping was applied due to a limit of using an equation of state that is required for dynamic stopping calculation.

## Data Availability

The datasets generated during and/or analyzed during the current study are available on reasonable request to F. N. Beg (fbeg@ucsd.edu) and C. McGuffey (cmcguffey@ucsd.edu).
